# Glial regulation of critical period plasticity

**DOI:** 10.3389/fncel.2023.1247335

**Published:** 2023-11-16

**Authors:** Jacob Starkey, Eric J. Horstick, Sarah D. Ackerman

**Affiliations:** ^1^Department of Biology, West Virginia University, Morgantown, WV, United States; ^2^Department of Neuroscience, West Virginia University, Morgantown, WV, United States; ^3^Department of Pathology and Immunology, Brain Immunology and Glia Center, Washington University School of Medicine, St. Louis, MO, United States

**Keywords:** critical period plasticity, astrocyte, oligodendrocyte, microglia, pruning, extracellular matrix, E/I balance

## Abstract

Animal behavior, from simple to complex, is dependent on the faithful wiring of neurons into functional neural circuits. Neural circuits undergo dramatic experience-dependent remodeling during brief developmental windows called critical periods. Environmental experience during critical periods of plasticity produces sustained changes to circuit function and behavior. Precocious critical period closure is linked to autism spectrum disorders, whereas extended synaptic remodeling is thought to underlie circuit dysfunction in schizophrenia. Thus, resolving the mechanisms that instruct critical period timing is important to our understanding of neurodevelopmental disorders. Control of critical period timing is modulated by neuron-intrinsic cues, yet recent data suggest that some determinants are derived from neighboring glial cells (astrocytes, microglia, and oligodendrocytes). As glia make up 50% of the human brain, understanding how these diverse cells communicate with neurons and with each other to sculpt neural plasticity, especially during specialized critical periods, is essential to our fundamental understanding of circuit development and maintenance.

## 1. Introduction

### 1.1. Discovering critical period plasticity

Critical periods are brief windows when sensory experience induces structural and functional plasticity at neuronal synapses to facilitate long-term changes in brain function and behavior. The first description of experience-dependent synaptic remodeling in the brain was published in the classic Wiesel and Hubel (1963) study of the cat visual cortex. In brief, the authors determined that occluding visual input to one eye (e.g., monocular deprivation) resulted in weakening of synapses downstream of the occluded eye, along with strengthening of corresponding synapses downstream of the open eye— a process they termed *ocular dominance*. This structural remodeling resulted in sustained changes to visual acuity. Importantly, ocular dominance plasticity was limited to a narrow developmental window (critical period) around eye opening, after which visual experience had limited impact of circuit structure/function (Presson and Gordon, 1979; LeVay et al., 1980; Maffei et al., 1992; Fagiolini et al., 1994; Gordon and Stryker, 1996; Levelt and Hübener, 2012; Chen et al., 2014). As neuronal plasticity is the basis of learning and memory, understanding why and how plasticity is developmentally restricted has fascinated neuroscientists for decades.

### 1.2. Classic determinants of critical period timing

While the mechanisms that instruct critical period timing and expression have been best studied in the visual cortex, work in other systems suggest broad conservation of these pathways across other circuits and species (Reh et al., 2020; Ackerman et al., 2021; Hageter et al., 2023). The onset of critical period plasticity requires the introduction of inhibitory circuit elements. In mammals, critical period opening in the cortex requires the maturation of fast-spiking, GABAergic, parvalbumin + interneurons (PV cells; reviewed in Takesian and Hensch, 2013). Indeed, critical period onset can be accelerated by precocious inhibitory circuit maturation (Hanover et al., 1999; Huang et al., 1999; Di Cristo et al., 2001; Sugiyama et al., 2008), or by premature, pharmacological activation of GABA_A_ receptors with benzodiazepines (Hensch et al., 1998; Fagiolini and Hensch, 2000; Fagiolini et al., 2003; Iwai et al., 2003). By contrast, failed maturation of inhibitory neurons via deletion of *Gad65*, an enzyme that is necessary for production of GABA, can permanently delay the visual critical period (Hensch et al., 1998; Fagiolini and Hensch, 2000). Thus, excitatory/inhibitory (E/I) balance is an important determinant of critical period onset.

Beyond expression of GABA, maturation of PV cells is accompanied by the development of a unique extracellular scaffold called perineuronal nets. Perineuronal nets are a mesh-like structure composed of chondroitin sulfate proteoglycans (CSPGs) and other extracellular matrix components that encase the cell bodies and proximate neurites of PV cells. CSPGs are known molecular brakes that prevent remodeling and repair of the injured central nervous system (CNS, Levine, 1994; McKeon et al., 1999; Asher et al., 2000; Bradbury et al., 2002). In development, application of chondroitinase-ABC, an enzyme that degrades CSPGs, can reopen plasticity in the adult visual system and basolateral amygdala (Pizzorusso et al., 2002, 2006; Gogolla et al., 2009). Interestingly, experimental conditions that delay critical period closure, such as developmental sensory deprivation, impair the development of perineuronal nets (Sur et al., 1988; McRae et al., 2007; Balmer et al., 2009; Ye and Miao, 2013). Thus, perineuronal net maturation is timed by environmental experience, which in turn functions as a timer to limit activity-dependent remodeling of the underlying neural circuit.

### 1.3. Glial regulation of neural plasticity

**FIGURE 1:**
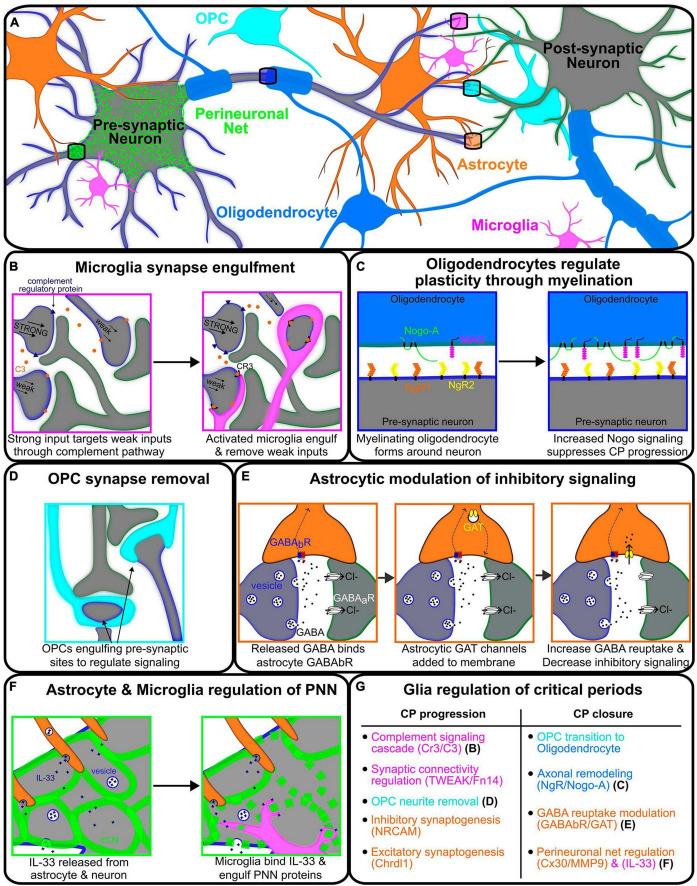
Glial modulation of signaling between two neurons regulating critical periods associated plasticity. **(A)** Representative illustration of various glial cells regulating the signaling between pre- (gray-blue) and post-synaptic (gray-green) neurons. **(B)** Neuron with the strongest synaptic input creating complement regulating proteins (blue) and releasing C3 proteins (orange) onto weaker inputs, thus signaling to microglia for engulfment and removal. **(C)** Oligodendrocyte (blue) myelination wrapping the axon of pre-synaptic neurons (gray-blue) and communicating through NgR/Nogo-A signaling. Oligodendrocyte Nogo-A (green) and MAG (magenta) are upregulated through critical period with increased binding to neuronal NgR1/2 (orange/yellow) to solidify projection and decrease plasticity. **(D)** Oligodendrocyte precursor cell (OPC, cyan) regulating neuronal signaling via phagocytosis of the pre-synaptic axon terminals. **(E)** Astrocyte modulating the strength of inhibitory signaling between neurons by utilizing GABA_B_R (blue-red) to detect GABA release and upregulate GAT (yellow) channels to uptake GABA, reducing critical period associated inhibitory signaling. **(F)** Astrocyte (orange) and microglia (magenta) regulating the formation of the perineuronal net in the extracellular matrix (ECM) around the pre-synaptic neuron. IL-33 is released from the astrocyte and neuron, signaling the microglia to break down the ECM and thus, perineuronal net formation. **(G)** Table describing the processes of glial regulation of critical period progression and closure for microglia (magenta), astrocytes (orange), OPCs (cyan), and oligodendrocytes (blue).

While much of the critical period field has focused on neuronal mechanisms of critical period timing, such as circuit inhibition, neurons do not function in isolation. A prominent proportion of brain mass is composed of non-neuronal cells called glia, including microglia, as well as the macroglia (astrocytes and oligodendrocytes). Changes in glial development strongly impact neural circuit function, from relatively simple invertebrates through humans (Lago-Baldaia et al., 2020). In this mini review, we discuss how diverse glial cell types communicate with neurons and with each other to mediate critical period plasticity (Figure 1). Finally, we highlight a growing body of evidence that glia are essential regulators of critical period timing. Together, these studies highlight the importance of considering non-neuronal cells in shaping experience-dependent circuit remodeling.

## 2. Glia as mediators of critical period plasticity via

### 2.1. Synaptic pruning

In development, neurons make more synapses than they ultimately require. During critical periods of plasticity, ectopic synaptic connections are then pruned in an activity-dependent manner, a process that is heavily regulated by all glial types (Allen and Eroglu, 2017; Wilton et al., 2019; Faust et al., 2021; Auguste et al., 2022a; Xiao et al., 2022). Microglia, the resident immune cells of the CNS, were long thought to act only as sentinels of pathology. While there are some neuron-intrinsic mechanisms of pruning (Parrish et al., 2007; Riccomagno and Kolodkin, 2015; Yaron and Schuldiner, 2016), the presence of professional phagocytes in the brain prompted an important question: can microglia phagocytose neuronal materials in a homeostatic context? Indeed, the last 10-years revealed many essential roles for microglia in sculpting neural circuit structure (reviewed in Faust et al., 2021). This is particularly well-studied studied in the developing visual circuit, which we will focus on here for simplicity, though we note that activity-dependent synaptic pruning by microglia is physiologically relevant across the brain (Paolicelli et al., 2011; Kopec et al., 2018; Vainchtein et al., 2018; Gunner et al., 2019; VanRyzin et al., 2020; Faust et al., 2021; Dayananda et al., 2023).

In a series of landmark studies from the Barres and Stevens labs, the authors demonstrated that neuronal activity drives microglial-refinement of retinogeniculate synapses during an early postnatal “pruning period” (Stevens et al., 2007; Schafer et al., 2012). In mammals, retinal ganglion cells (RGCs) send both contralateral and ipsilateral projections to the dorsal lateral geniculate nucleus (dLGN); thus, each dLGN receives connections from both eyes. During development, left and right eye RGCs form supernumerary synapses on relay neurons with overlapping dendritic territories within the dLGN. Through high resolution imaging, microglia were shown to position themselves near RGC > dLGN synapses at postnatal (P) day 5 (peak refinement) in mouse, and synaptic material could be found inside microglial processes. The authors then showed that microglial engulfment of RGC synapses is activity-dependent, requires the complement signaling cascade (CR3/C3; Figures 1A, B), and that impairing complement-dependent synaptic pruning permanently altered RGC > dLGN connectivity (Stevens et al., 2007; Schafer et al., 2012). Thus, microglia can regulate activity-dependent refinement of synaptic connections in the developing (P5) mouse brain.

While the pruning period peaks prior to eye opening, and is thus outside the context of the classic visual critical period defined by Hubel and Weisel (peaks at ∼P28 in mouse; Tremblay et al., 2010), these initial studies laid the groundwork for numerous follow up studies on microglial behavior in other developmental contexts, including ocular dominance. During monocular deprivation, microglia become hyper-ramified and exhibit increased association with neuronal synapses (Tremblay et al., 2010). Recent data suggests that activity-dependent changes in microglial morphology and function are, in part, due to P2Y12 purinergic receptor signaling. P2Y12 is specifically expressed in homeostatic microglia, and changes in P2Y12 signaling are accompanied by reduced microglia-synapse association and reduced ocular dominance plasticity (Sipe et al., 2016). These data suggest that monocular deprivation induces microglial engulfment of inactive synapses, as observed during the “pruning period” in the retinogeniculate system (Stevens et al., 2007; Schafer et al., 2012). To test this, Ma et al. (2020) performed pharmacological depletion of microglia using a colony-stimulating factor 1 receptor inhibitor PLX3397 during the visual critical period (P14 through P28). Here, they found increased spine density on L5 pyramidal (excitatory) neurons, presumably due to lack of microglial synaptic pruning. Accordingly, ocular dominance plasticity was diminished during the visual critical period (Ma et al., 2020). Together, these data demonstrate the importance of microglial synaptic pruning for activity-dependent synapse refinement during the visual critical period. Interestingly, a recent study demonstrated that microglia can also regulate RGC > dLGN connectivity independent of their role in synaptic pruning via a TWEAK/Fn14 intercellular signaling axis (Cheadle et al., 2020), suggesting that deeper exploration of microglia-synapse communication is required to fully understand the role of microglia in experience-dependent synaptic remodeling.

### 2.2. Modulating the extracellular environment

While microglia are better known for their role in synaptic pruning, recent data indicate that microglia can also regulate the structure of the extracellular matrix (ECM). The ECM makes up 20% of the volume of the human brain, serving many diverse roles including cell signaling, barrier functions, and structural support (Nicholson and Syková, 1998; Crapser et al., 2021). Glial cells secrete both ECM and ECM-modulating enzymes, including MMPS (matrix metalloproteases) and ADAMs (a disintegrin and metalloprotease), and recent data suggests that glial-modulation of the ECM can have a profound effect on synapse stability in development (Desjardins et al., 1992; Crapser et al., 2021; Tewari et al., 2022). As microglia-ECM dynamics are thoroughly reviewed elsewhere (Crapser et al., 2021), here we will focus on a set of studies that beautifully highlight how diverse glial cell types communicate with one another, and the environment, to shape critical period plasticity. Recent work from the Molofsky lab identified astrocyte-derived IL-33 as a key modulator of microglial phagocytosis. Loss of *Il33* depressed microglial engulfment of excitatory synapses in the spinal cord and thalamus, leading to an accumulation of synapses and impaired circuit function (Vainchtein et al., 2018; Figure 1F). More recently, they went on to show that in adulthood, *Il33* is strongly expressed in hippocampal neurons. Interestingly, neuron-derived IL-33 drove microglial engulfment of the ECM, which was required for experience-dependent synaptic remodeling and memory consolidation at adult stages (Nguyen et al., 2020). Thus, microglia are essential regulators of activity-dependent synaptic remodeling in development and in the adult brain.

### 2.3. Axonal remodeling

Beyond synaptic remodeling, glial cells can both mediate and suppress larger-scale structural remodeling of neurons during developmental critical periods. Oligodendrocytes are the myelinating glia of the CNS, iteratively wrapping axons to form the myelin sheath, an insulating membrane required for rapid action potential propagation. Although oligodendrocytes derive from oligodendrocyte precursor cells (OPCs), OPCs are abundant in the CNS well into adulthood (Dawson et al., 2003), and increasing evidence suggests that OPCs have many independent functions apart from their role as precursor cells (Lin and Bergles, 2004; Fernandez-Castaneda and Gaultier, 2016; Auguste et al., 2022a; Li et al., 2022; Xiao et al., 2022). A recent study in zebrafish identified a novel role for OPCs in sculpting the developing visual circuit (Xiao et al., 2022; Figure 1D). Intriguingly, the authors found that the ratio of OPCs to myelinating glia differed across brain regions, with the optic tectum showing relatively little myelin even into juvenile stages (14 days post-fertilization), though OPCs were abundant. This led the authors to question: what is the role of OPCs in the optic tectum, if not to generate myelinating oligodendrocytes? The appearance of OPCs coincided with the arrival of RGC axons within the tectum. To test the functional significance of this association, the authors genetically ablated OPCs using the inducible nitroreductase-mediated cell ablation system. In brief, nitroreductase is an enzyme that reduces the antibiotic metronidazole into a cytotoxic compound, resulting in cell death (Curado et al., 2008). Thus, using an OPC-specific nitroreductase transgenic line, the authors were able to genetically ablate OPCs with cell type-specific, temporal precision. Interestingly, removal of OPCs during activity-dependent circuit refinement (7 days post-fertilization) resulted in ectopic RGC neurites and altered visual processing (Xiao et al., 2022). A similar role was then described for OPCs in pruning synapses in the developing mouse visual system (Auguste et al., 2022b). Thus, OPCs mediate experience-dependent circuit remodeling in development (Figure 1D).

## 3. Glia as mediators of critical period timing

Recent data indicate that apart from mediating plasticity *within* critical periods, glia can non-autonomously regulate critical period timing. These studies shed new light on classic mediators of critical period closure (E/I balance and ECM cues) and unveil new pathways that warrant further investigation (summarized in Figure 1G).

### 3.1. E/I balance in developing neural circuits

It is well-known that circuit inhibition is a key determinant of critical period timing, as altering the level of GABA or disrupting the maturation of key inhibitory neurons can delay critical period closure (Hensch et al., 1998; Fagiolini and Hensch, 2000). Astrocytes are the most abundant glial cell type in the brain, where they extend many fine processes to simultaneously interact with thousands to millions of neuronal synapses as part of the tripartite synapse (reviewed in Allen and Eroglu, 2017; Perez-Catalan et al., 2021). One of the most important roles of astrocytes at the synapse is regulating neurotransmitter turnover, and in turn, E/I balance. To do this, astrocytes express both excitatory and inhibitory neurotransmitter transporters. Astrocyte-specific GABA transporters (GATs) allow for the uptake of GABA from local synapses to modulate signaling (Shigetomi et al., 2011; Muthukumar et al., 2014). In addition, astrocyte-specific metabotropic GABA-B receptors act as regulators of GABA reuptake (Muthukumar et al., 2014; Figure 1E). The levels of GAT expression is critical for GABAergic synapse function, as mouse GAT-1 mutants show a significant decrease in GABA reuptake by astrocytes in the cortex and thalamus, resulting in epilepsy-like seizures (Mermer et al., 2022). *Drosophila* mutants for the sole GABA transporter Gat are embryonic lethal (Stork et al., 2014). Thus, astrocyte modulation of local and global neurotransmitter levels is essential for circuit function and animal survival. As E/I balance is a key determinant of critical period timing, astrocytes are well-placed to sculpt local and global patterns of plasticity.

The first line of evidence that astrocytes shape critical period timing was published in Müller and Best (1989), when demonstrated that implantation of immature astrocytes into the adult cat visual cortex was sufficient to reopen ocular dominance plasticity, which was recently validated in mouse (Ribot et al., 2021). These data suggested that immature astrocytes create an environment that is permissive of critical period plasticity. In search of the elusive cue released by immature astrocytes to promote plasticity, Ribot et al. leveraged mouse genetics to delay astrocyte maturation. They found that astrocyte maturation coincides with a switch from a mitotic state to a state that prioritizes cell-cell communication. Thus, to perturb astrocyte maturation, they performed astrocyte knockdown (KD) of the gap-junction channel subunit connexin 30 (Cx30), a protein that is known to regulate astrocyte to astrocyte signaling. They found that astrocyte KD of Cx30 reopened ocular dominance plasticity in adult mice due to altered PV neuron maturation. The authors observed a significant reduction in both miniature excitatory and inhibitory post-synaptic currents (mEPSCs and mIPSCs) from V1 pyramidal neurons, with a greater reduction observed in mIPSCs. Thus, changes in astrocyte Cx30 delayed the maturation of inhibitory circuits required for critical period closure. To further address how astrocytes might modulate the timing of critical period plasticity, a recent study leveraged *Drosophila* to screen for astrocyte-derived cues that drive critical period closure. During this critical period, changes in motor neuron activity shaped the numbers and distribution of excitatory and inhibitory inputs onto motor dendrites in a homeostatic manner (Ackerman et al., 2021). Like sensory systems (Müller and Best, 1989; Ribot et al., 2021), the authors found that the motor critical period is terminated by astrocyte maturation, and that astrocyte-specific ablation delayed the closure of the motor critical period. Moreover, they found that KD of the astrocyte GABA transporter (Gat) abolished the ability of astrocytes to uptake GABA, disrupted E/I balance, and extended plasticity (Ackerman et al., 2021).

While the above studies demonstrate that astrocytes modulate synaptic function and thus critical period timing, it is important to note that a primary role of astrocytes is to regulate both excitatory and inhibitory synaptogenesis (Ullian et al., 2004; Kucukdereli et al., 2011; Muthukumar et al., 2014; Blanco-Suarez et al., 2018; Takano et al., 2020), which also influences E/I balance across circuits. For example, astrocytes express neuronal cell adhesion molecule (NRCAM), which complexes with gephyrin to stabilize GABAergic synapses (Takano et al., 2020), a key determinant of critical period timing (Hensch et al., 1998; Fagiolini and Hensch, 2000). Interestingly, a recent study demonstrated that astrocyte-specific deletion of chordin like 1 (Chrdl1), which regulates excitatory synapse maturation, was sufficient to extend ocular dominance plasticity to adult stages (Blanco-Suarez et al., 2018; Figure 1G). These data indicated that astrocytes time synapse maturation to instruct critical period timing.

### 3.2. ECM structure and composition

In addition to E/I balance, maturation of the ECM is a primary driver of critical period closure (Reh et al., 2020). As noted above, immature astrocytes are enriched in the visual cortex during the critical period (Müller and Best, 1989; Ribot et al., 2021), and astrocyte maturation instructs critical period closure. In addition to changes in E/I balance, Reh et al. (2020) found that Cx30 functions to regulate ECM maturation around developing PV neurons. Although Cx30 is better known for mediating astrocyte-astrocyte communication as a gap junction protein, they found that Cx30 signals through RhoA/ROCK to suppress expression of MMP9, a matrix metalloprotease that degrades perineuronal nets. Thus, astrocyte-specific KD of Cx30 increased MMP9 expression and prevented perineuronal net formation, which in turn delayed PV neuron maturation and extended ocular dominance plasticity (Figure 1G). Cortical engraftment of mature astrocytes without Cx30 was sufficient to enhance visual plasticity in adult mice. These data support Müller and Best’s seminal finding that immature astrocytes can reopen critical period plasticity, and identify Cx30 as a key intervention point. Of note, astrocyte-specific KD of ECM proteins was also sufficient to extend the critical period of motor circuit plasticity in *Drosophila* (Ackerman et al., 2021); thus, astrocyte regulation of the extracellular environment is an evolutionarily conserved mechanism that drives critical period closure.

### 3.3. Myelination and critical period closure

Recent data from mouse and from *Drosophila* indicate that the timing of astrocyte maturation can predict critical period closure (Ackerman et al., 2021; Ribot et al., 2021). Astrocytes and oligodendrocytes are both born late in embryonic development, and oligodendrocyte maturation similarly mirrors the transition from critical period plasticity to stability. Indeed, a recent study found that blocking oligodendrocyte maturation was sufficient to extend ocular dominance plasticity (Xin et al., 2023). While astrocytes mediate critical period closure through modulation of E/I balance and ECM composition, oligodendrocytes seem to function in parallel to ensure critical period closure. In a landmark study, McGee et al. (2005) demonstrated that the myelin proteins MAG and Nogo-A increase in abundance during the visual critical period. As Nogo Receptor (NgR) had previously been linked to suppressed CNS regeneration (GrandPré et al., 2002; Kim et al., 2003), the authors questioned whether NgR might similarly suppress ocular dominance plasticity. Indeed, monocular deprivation in adult *NgR* knockout mice (postnatal day 45–49) resulted in an ocular dominance shift that is not observed in wildtype controls; importantly, the Nogo-A mutant mice phenocopy this result. Together, these data indicate that ocular dominance plasticity is extended beyond development in the absence of NgR/Nogo-A signaling between neurons and myelinating oligodendrocytes (McGee et al., 2005). Of note, while NgR signaling regulates structural remodeling of neurons following injury (GrandPré et al., 2002; Kim et al., 2003), NgR signaling appears to limit developmental plasticity through timing PV cell maturation and E/I balance (Stephany et al., 2016). Thus, immature oligodendrocytes (e.g., OPCs, see section “2.3. Axonal remodeling”) promote critical period plasticity (Figure 1D), whereas myelinating oligodendrocytes are key regulators of critical period closure (Figure 1C). As neural activity can drive OPC differentiation and myelination (Hill et al., 2014; Monje, 2018), neurons and developing oligodendrocytes reciprocally communicate to set critical period closure.

## 4. Discussion

Development is an especially important time in an animal’s life. Social, emotional, and environmental experiences during key developmental critical periods have dramatic and long-lasting consequences to brain function. Thus, understanding the mechanisms that set critical period timing will impact how we understand human behavior and disease. It is increasingly clear that diverse glial types (OPCs, oligodendrocytes, astrocytes, and microglia) shape both the expression and timing of neural plasticity. In other words, neurons may be the musicians, but glia may very well be in the conductor’s seat.

## Author contributions

SA and EH conceived the topic. SA and JS co-wrote the manuscript. JS generated the associated figure. All authors read and approved the manuscript before submission.

## References

[B1] Ackerman S. Perez-Catalan N. Freeman M. Doe C. (2021). Astrocytes close a motor circuit critical period. *Nature* 592 414–420. 10.1038/s41586-021-03441-2 33828296 PMC9901311

[B2] Allen N. Eroglu C. (2017). Cell biology of astrocyte-synapse interactions. *Neuron* 96 697–708. 10.1016/j.neuron.2017.09.056 29096081 PMC5687890

[B3] Asher R. Morgenstern D. Fidler P. Adcock K. Oohira A. Braistead J. (2000). Neurocan is upregulated in injured brain and in cytokine-treated astrocytes. *J. Neurosci.* 20 2427–2438. 10.1523/JNEUROSCI.20-07-02427.2000 10729323 PMC6772249

[B4] Auguste Y. Ferro A. Kahng J. Xavier A. Dixon J. Vrudhula U. (2022a). Oligodendrocyte precursor cells engulf synapses during circuit remodeling in mice. *Nat. Neurosci.* 25 1273–1278. 10.1038/s41593-022-01170-x 36171430 PMC9534756

[B5] Auguste Y. Ferro A. Kahng J. Xavier A. Dixon J. Vrudhula U. (2022b). Publisher Correction: Oligodendrocyte precursor cells engulf synapses during circuit remodeling in mice. *Nat. Neurosci.* 25:1735. 10.1038/s41593-022-01209-z 36344700 PMC9708582

[B6] Balmer T. Carels V. Frisch J. Nick T. (2009). Modulation of perineuronal nets and parvalbumin with developmental song learning. *J. Neurosci.* 29 12878–12885. 10.1523/JNEUROSCI.2974-09.2009 19828802 PMC2769505

[B7] Blanco-Suarez E. Liu T. Kopelevich A. Allen N. (2018). Astrocyte-secreted chordin-like 1 drives synapse maturation and limits plasticity by increasing synaptic GluA2 AMPA receptors. *Neuron* 100 1116–1132.e13. 10.1016/j.neuron.2018.09.043 30344043 PMC6382071

[B8] Bradbury E. Moon L. Popat R. King V. Bennett G. Patel P. (2002). Chondroitinase ABC promotes functional recovery after spinal cord injury. *Nature* 416 636–640. 10.1038/416636a 11948352

[B9] Cheadle L. Rivera S. Phelps J. Ennis K. Stevens B. Burkly L. (2020). Sensory experience engages microglia to shape neural connectivity through a non-phagocytic mechanism. *Neuron* 108 451–468.e9. 10.1016/j.neuron.2020.08.002 32931754 PMC7666095

[B10] Chen X. Rasch M. Chen G. Ye C. Wu S. Zhang X. (2014). Binocular input coincidence mediates critical period plasticity in the mouse primary visual cortex. *J. Neurosci.* 34 2940–2955. 10.1523/JNEUROSCI.2640-13.2014 24553935 PMC6608519

[B11] Crapser J. Arreola M. Tsourmas K. Green K. (2021). Microglia as hackers of the matrix: Sculpting synapses and the extracellular space. *Cell Mol. Immunol.* 18 2472–2488. 10.1038/s41423-021-00751-3 34413489 PMC8546068

[B12] Curado S. Stainier D. Anderson R. (2008). Nitroreductase-mediated cell/tissue ablation in zebrafish: A spatially and temporally controlled ablation method with applications in developmental and regeneration studies. *Nat. Protoc.* 3 948–954. 10.1038/nprot.2008.58 18536643 PMC2705989

[B13] Dawson M. Polito A. Levine J. Reynolds R. (2003). NG2-expressing glial progenitor cells: An abundant and widespread population of cycling cells in the adult rat CNS. *Mol. Cell Neurosci.* 24 476–488. 10.1016/s1044-7431(03)00210-0 14572468

[B14] Dayananda K. Ahmed S. Wang D. Polis B. Islam R. Kaffman A. (2023). Early life stress impairs synaptic pruning in the developing hippocampus. *Brain Behav. Immun.* 107 16–31. 10.1016/j.bbi.2022.09.014 36174883 PMC10497209

[B15] Desjardins R. Fournier M. Denizeau F. Krzystyniak K. (1992). Immunosuppression by chronic exposure to N-nitrosodimethylamine (NDMA) in mice. *J. Toxicol. Environ. Health* 37 351–361. 10.1080/15287399209531676 1433375

[B16] Di Cristo G. Berardi N. Cancedda L. Pizzorusso T. Putignano E. Ratto G. (2001). Requirement of ERK activation for visual cortical plasticity. *Science* 292 2337–2340. 10.1126/science.1059075 11423664

[B17] Fagiolini M. Hensch T. (2000). Inhibitory threshold for critical-period activation in primary visual cortex. *Nature* 404 183–186. 10.1038/35004582 10724170

[B18] Fagiolini M. Katagiri H. Miyamoto H. Mori H. Grant S. Mishina M. (2003). Separable features of visual cortical plasticity revealed by N-methyl-D-aspartate receptor 2A signaling. *Proc. Natl. Acad. Sci. U. S. A.* 100 2854–2859. 10.1073/pnas.0536089100 12591944 PMC151430

[B19] Fagiolini M. Pizzorusso T. Berardi N. Domenici L. Maffei L. (1994). Functional postnatal development of the rat primary visual cortex and the role of visual experience: Dark rearing and monocular deprivation. *Vision Res.* 34 709–720. 10.1016/0042-6989(94)90210-0 8160387

[B20] Faust T. Gunner G. Schafer D. (2021). Mechanisms governing activity-dependent synaptic pruning in the developing mammalian CNS. *Nat. Rev. Neurosci.* 22 657–673. 10.1038/s41583-021-00507-y 34545240 PMC8541743

[B21] Fernandez-Castaneda A. Gaultier A. (2016). Adult oligodendrocyte progenitor cells - Multifaceted regulators of the CNS in health and disease. *Brain Behav. Immun.* 57 1–7. 10.1016/j.bbi.2016.01.005 26796621 PMC4940337

[B22] Gogolla N. Caroni P. Lüthi A. Herry C. (2009). Perineuronal nets protect fear memories from erasure. *Science* 325 1258–1261. 10.1126/science.1174146 19729657

[B23] Gordon J. Stryker M. (1996). Experience-dependent plasticity of binocular responses in the primary visual cortex of the mouse. *J. Neurosci.* 16 3274–3286. 10.1523/JNEUROSCI.16-10-03274.1996 8627365 PMC6579137

[B24] GrandPré T. Li S. Strittmatter S. (2002). Nogo-66 receptor antagonist peptide promotes axonal regeneration. *Nature* 417 547–551. 10.1038/417547a 12037567

[B25] Gunner G. Cheadle L. Johnson K. Ayata P. Badimon A. Mondo E. (2019). Sensory lesioning induces microglial synapse elimination via ADAM10 and fractalkine signaling. *Nat. Neurosci.* 22 1075–1088. 10.1038/s41593-019-0419-y 31209379 PMC6596419

[B26] Hageter J. Starkey J. Horstick E. (2023). Thalamic regulation of a visual critical period and motor behavior. *Cell Rep.* 42:112287. 10.1016/j.celrep.2023.112287 36952349 PMC10514242

[B27] Hanover J. Huang Z. Tonegawa S. Stryker M. (1999). Brain-derived neurotrophic factor overexpression induces precocious critical period in mouse visual cortex. *J. Neurosci.* 19:RC40. 10.1523/JNEUROSCI.19-22-j0003.1999 10559430 PMC2424259

[B28] Hensch T. Fagiolini M. Mataga N. Stryker M. Baekkeskov S. Kash S. (1998). Local GABA circuit control of experience-dependent plasticity in developing visual cortex. *Science* 282 1504–1508. 10.1126/science.282.5393.1504 9822384 PMC2851625

[B29] Hill R. Patel K. Goncalves C. Grutzendler J. Nishiyama A. (2014). Modulation of oligodendrocyte generation during a critical temporal window after NG2 cell division. *Nat. Neurosci.* 17 1518–1527. 10.1038/nn.3815 25262495 PMC4275302

[B30] Huang Z. Kirkwood A. Pizzorusso T. Porciatti V. Morales B. Bear M. (1999). BDNF regulates the maturation of inhibition and the critical period of plasticity in mouse visual cortex. *Cell* 98 739–755. 10.1016/s0092-8674(00)81509-3 10499792

[B31] Iwai Y. Fagiolini M. Obata K. Hensch T. (2003). Rapid critical period induction by tonic inhibition in visual cortex. *J. Neurosci.* 23 6695–6702. 10.1523/JNEUROSCI.23-17-06695.2003 12890762 PMC6740711

[B32] Kim J. Li S. GrandPré T. Qiu D. Strittmatter S. (2003). Axon regeneration in young adult mice lacking Nogo-A/B. *Neuron* 38 187–199. 10.1016/s0896-6273(03)00147-8 12718854

[B33] Kopec A. Smith C. Ayre N. Sweat S. Bilbo S. (2018). Microglial dopamine receptor elimination defines sex-specific nucleus accumbens development and social behavior in adolescent rats. *Nat. Commun.* 9:3769. 10.1038/s41467-018-06118-z 30254300 PMC6156594

[B34] Kucukdereli H. Allen N. Lee A. Feng A. Ozlu M. Conatser L. (2011). Control of excitatory CNS synaptogenesis by astrocyte-secreted proteins Hevin and SPARC. *Proc. Natl. Acad. Sci. U. S. A.* 108 E440–E449. 10.1073/pnas.1104977108 21788491 PMC3156217

[B35] Lago-Baldaia I. Fernandes V. Ackerman S. (2020). More than mortar: Glia as architects of nervous system development and disease. *Front. Cell Dev. Biol.* 8:611269. 10.3389/fcell.2020.611269 33381506 PMC7767919

[B36] LeVay S. Wiesel T. Hubel D. (1980). The development of ocular dominance columns in normal and visually deprived monkeys. *J. Comp. Neurol.* 191 1–51. 10.1002/cne.901910102 6772696

[B37] Levelt C. Hübener M. (2012). Critical-period plasticity in the visual cortex. *Annu. Rev. Neurosci.* 35 309–330. 10.1146/annurev-neuro-061010-113813 22462544

[B38] Levine J. (1994). Increased expression of the NG2 chondroitin-sulfate proteoglycan after brain injury. *J. Neurosci.* 14 4716–4730. 10.1523/JNEUROSCI.14-08-04716.1994 8046446 PMC6577168

[B39] Li J. Miramontes T. Czopka T. Monk K. (2022). Synapses and Ca2+ activity in oligodendrocyte precursor cells predict where myelin sheaths form. *Biorxiv* [Preprint]. 10.1101/2022.03.18.484955 38216650

[B40] Lin S. Bergles D. (2004). Synaptic signaling between GABAergic interneurons and oligodendrocyte precursor cells in the hippocampus. *Nat. Neurosci.* 7 24–32. 10.1038/nn1162 14661022

[B41] Ma X. Chen K. Cui Y. Huang G. Nehme A. Zhang L. (2020). Depletion of microglia in developing cortical circuits reveals its critical role in glutamatergic synapse development, functional connectivity, and critical period plasticity. *J. Neurosci. Res.* 98:1968. 10.1002/jnr.24641 32594561

[B42] Maffei L. Berardi N. Domenici L. Parisi V. Pizzorusso T. (1992). Nerve growth factor (NGF) prevents the shift in ocular dominance distribution of visual cortical neurons in monocularly deprived rats. *J. Neurosci.* 12 4651–4662. 10.1523/JNEUROSCI.12-12-04651.1992 1334503 PMC6575769

[B43] McGee A. Yang Y. Fischer Q. Daw N. Strittmatter S. (2005). Experience-driven plasticity of visual cortex limited by myelin and Nogo receptor. *Science* 309 2222–2226. 10.1126/science.1114362 16195464 PMC2856689

[B44] McKeon R. Jurynec M. Buck C. (1999). The chondroitin sulfate proteoglycans neurocan and phosphacan are expressed by reactive astrocytes in the chronic CNS glial scar. *J. Neurosci.* 19 10778–10788. 10.1523/JNEUROSCI.19-24-10778.1999 10594061 PMC6784959

[B45] McRae P. Rocco M. Kelly G. Brumberg J. Matthews R. (2007). Sensory deprivation alters aggrecan and perineuronal net expression in the mouse barrel cortex. *J. Neurosci.* 27 5405–5413. 10.1523/JNEUROSCI.5425-06.2007 17507562 PMC6672348

[B46] Mermer F. Poliquin S. Zhou S. Wang X. Ding Y. Yin F. (2022). Astrocytic GABA transporter 1 deficit in novel SLC6A1 variants mediated epilepsy: Connected from protein destabilization to seizures in mice and humans. *Neurobiol. Dis.* 172:105810. 10.1016/j.nbd.2022.105810 35840120 PMC9472560

[B47] Monje M. (2018). Myelin plasticity and nervous system function. *Annu. Rev. Neurosci.* 41 61–76. 10.1146/annurev-neuro-080317-061853 29986163

[B48] Müller C. Best J. (1989). Ocular dominance plasticity in adult cat visual cortex after transplantation of cultured astrocytes. *Nature* 342 427–430. 10.1038/342427a0 2586611

[B49] Muthukumar A. Stork T. Freeman M. (2014). Activity-dependent regulation of astrocyte GAT levels during synaptogenesis. *Nat. Neurosci.* 17 1340–1350. 10.1038/nn.3791 25151265 PMC4176984

[B50] Nguyen P. Dorman L. Pan S. Vainchtein I. Han R. Nakao-Inoue H. (2020). Microglial remodeling of the extracellular matrix promotes synapse plasticity. *Cell* 182 388–403.e15. 10.1016/j.cell.2020.05.050 32615087 PMC7497728

[B51] Nicholson C. Syková E. (1998). Extracellular space structure revealed by diffusion analysis. *Trends Neurosci.* 21 207–215. 10.1016/s0166-2236(98)01261-2 9610885

[B52] Paolicelli R. Bolasco G. Pagani F. Maggi L. Scianni M. Panzanelli P. (2011). Synaptic pruning by microglia is necessary for normal brain development. *Science* 333 1456–1458. 10.1126/science.1202529 21778362

[B53] Parrish J. Emoto K. Kim M. Jan Y. (2007). Mechanisms that regulate establishment, maintenance, and remodeling of dendritic fields. *Annu. Rev. Neurosci.* 30 399–423. 10.1146/annurev.neuro.29.051605.112907 17378766

[B54] Perez-Catalan N. Doe C. Ackerman S. (2021). The role of astrocyte-mediated plasticity in neural circuit development and function. *Neural Dev.* 16:1. 10.1186/s13064-020-00151-9 33413602 PMC7789420

[B55] Pizzorusso T. Medini P. Berardi N. Chierzi S. Fawcett J. Maffei L. (2002). Reactivation of ocular dominance plasticity in the adult visual cortex. *Science* 298 1248–1251. 10.1126/science.1072699 12424383

[B56] Pizzorusso T. Medini P. Landi S. Baldini S. Berardi N. Maffei L. (2006). Structural and functional recovery from early monocular deprivation in adult rats. *Proc. Natl. Acad. Sci. U. S. A.* 103 8517–8522. 10.1073/pnas.0602657103 16709670 PMC1482523

[B57] Presson J. Gordon B. (1979). Critical period and minimum exposure required for the effects of alternating monocular occlusion in cat visual cortex. *Vision Res.* 19 807–811. 10.1016/0042-6989(79)90157-3 483600

[B58] Reh R. Dias B. Nelson C. Kaufer D. Werker J. Kolb B. (2020). Critical period regulation across multiple timescales. *Proc. Natl. Acad. Sci. U. S. A.* 117 23242–23251. 10.1073/pnas.1820836117 32503914 PMC7519216

[B59] Ribot J. Breton R. Calvo C. Moulard J. Ezan P. Zapata J. (2021). Astrocytes close the mouse critical period for visual plasticity. *Science* 373 77–81. 10.1126/science.abf5273 34210880

[B60] Riccomagno M. Kolodkin A. (2015). Sculpting neural circuits by axon and dendrite pruning. *Annu. Rev. Cell Dev. Biol.* 31 779–805. 10.1146/annurev-cellbio-100913-013038 26436703 PMC4668927

[B61] Schafer D. Lehrman E. Kautzman A. Koyama R. Mardinly A. Yamasaki R. (2012). Microglia sculpt postnatal neural circuits in an activity and complement-dependent manner. *Neuron* 74 691–705. 10.1016/j.neuron.2012.03.026 22632727 PMC3528177

[B62] Shigetomi E. Tong X. Kwan K. Corey D. Khakh B. (2011). TRPA1 channels regulate astrocyte resting calcium and inhibitory synapse efficacy through GAT-3. *Nat. Neurosci.* 15 70–80. 10.1038/nn.3000 22158513 PMC3282183

[B63] Sipe G. Lowery R. Tremblay M. Kelly E. Lamantia C. Majewska A. (2016). Microglial P2Y12 is necessary for synaptic plasticity in mouse visual cortex. *Nat. Commun.* 7:10905. 10.1038/ncomms10905 26948129 PMC4786684

[B64] Stephany C. Ikrar T. Nguyen C. Xu X. McGee A. (2016). Nogo Receptor 1 confines a disinhibitory microcircuit to the critical period in visual cortex. *J. Neurosci.* 36 11006–11012. 10.1523/JNEUROSCI.0935-16.2016 27798181 PMC5098837

[B65] Stevens B. Allen N. Vazquez L. Howell G. Christopherson K. Nouri N. (2007). The classical complement cascade mediates CNS synapse elimination. *Cell* 131 1164–1178. 10.1016/j.cell.2007.10.036 18083105

[B66] Stork T. Sheehan A. Tasdemir-Yilmaz O. Freeman M. (2014). Neuron-glia interactions through the heartless FGF receptor signaling pathway mediate morphogenesis of drosophila astrocytes. *Neuron* 83 388–403. 10.1016/j.neuron.2014.06.026 25033182 PMC4124900

[B67] Sugiyama S. Di Nardo A. Aizawa S. Matsuo I. Volovitch M. Prochiantz A. (2008). Experience-dependent transfer of Otx2 homeoprotein into the visual cortex activates postnatal plasticity. *Cell* 134 508–520. 10.1016/j.cell.2008.05.054 18692473

[B68] Sur M. Frost D. Hockfield S. (1988). Expression of a surface-associated antigen on Y-cells in the cat lateral geniculate nucleus is regulated by visual experience. *J. Neurosci.* 8 874–882. 10.1523/JNEUROSCI.08-03-00874.1988 3346725 PMC6569223

[B69] Takano T. Wallace J. Baldwin K. Purkey A. Uezu A. Courtland J. (2020). Chemico-genetic discovery of astrocytic control of inhibition in vivo. *Nature* 588 296–302. 10.1038/s41586-020-2926-0 33177716 PMC8011649

[B70] Takesian A. Hensch T. (2013). Balancing plasticity/stability across brain development. *Prog. Brain Res.* 207 3–34. 10.1016/B978-0-444-63327-9.00001-1 24309249

[B71] Tewari B. Chaunsali L. Prim C. Sontheimer H. (2022). A glial perspective on the extracellular matrix and perineuronal net remodeling in the central nervous system. *Front. Cell Neurosci.* 16:1022754. 10.3389/fncel.2022.1022754 36339816 PMC9630365

[B72] Tremblay M. Lowery R. Majewska A. (2010). Microglial interactions with synapses are modulated by visual experience. *PLoS Biol.* 8:e1000527. 10.1371/journal.pbio.1000527 21072242 PMC2970556

[B73] Ullian E. Christopherson K. Barres B. (2004). Role for glia in synaptogenesis. *Glia* 47 209–216. 10.1002/glia.20082 15252809

[B74] Vainchtein I. Chin G. Cho F. Kelley K. Miller J. Chien E. (2018). Astrocyte-derived interleukin-33 promotes microglial synapse engulfment and neural circuit development. *Science* 359 1269–1273. 10.1126/science.aal3589 29420261 PMC6070131

[B75] VanRyzin J. Marquardt A. Pickett L. McCarthy M. (2020). Microglia and sexual differentiation of the developing brain: A focus on extrinsic factors. *Glia* 68 1100–1113. 10.1002/glia.23740 31691400 PMC8970113

[B76] Wiesel T. Hubel D. (1963). Single-cell responses in striate cortex of kittens deprived of vision in one eye. *J. Neurophysiol.* 26 1003–1017. 10.1152/jn.1963.26.6.1003 14084161

[B77] Wilton D. Dissing-Olesen L. Stevens B. (2019). Neuron-glia signaling in synapse elimination. *Annu. Rev. Neurosci.* 42 107–127. 10.1146/annurev-neuro-070918-050306 31283900

[B78] Xiao Y. Petrucco L. Hoodless L. Portugues R. Czopka T. (2022). Oligodendrocyte precursor cells sculpt the visual system by regulating axonal remodeling. *Nat. Neurosci.* 25 280–284. 10.1038/s41593-022-01023-7 35241802 PMC8904260

[B79] Xin W. Kaneko M. Roth R. H. Zhang A. Nocera S. Ding J. B. (2023). Adolescent oligodendrogenesis and myelination restrict experience-dependent neuronal plasticity in adult visual cortex. *bioRxiv [Preprint]*. 10.1101/2023.09.29.560231 37808666 PMC10557765

[B80] Yaron A. Schuldiner O. (2016). Common and divergent mechanisms in developmental neuronal remodeling and dying back neurodegeneration. *Curr. Biol.* 26 R628–R639. 10.1016/j.cub.2016.05.025 27404258 PMC5086086

[B81] Ye Q. Miao Q. (2013). Experience-dependent development of perineuronal nets and chondroitin sulfate proteoglycan receptors in mouse visual cortex. *Matrix Biol.* 32 352–363. 10.1016/j.matbio.2013.04.001 23597636

